# How I do it: Cochlear Osia 2 System surgery placement

**DOI:** 10.1007/s00405-022-07708-w

**Published:** 2022-10-22

**Authors:** Davide Soloperto, Virginia Dallari, Gabriele Molteni

**Affiliations:** Unit of Otorhinolaryngology, Head and Neck Department, University of Verona, Piazzale L.A. Scuro 10, 37134 Verona, Italy

**Keywords:** Osia, Otology, Conductive hearing loss, Bone conduction hearing, Speech perception

## Abstract

**Background:**

The Cochlear™ Osia^®^ 2 System is an active transcutaneous bone-anchored hearing implant with a newly developed piezoelectric transducer that is fixed to a titanium implant (BI300).

**Methods:**

It uses digital piezoelectric stimulation to bypass non-functional areas of the natural hearing system and send sound directly to the cochlea. This device is designed to meet the needs of patients with unilateral and bilateral conductive or mixed hearing loss and single-sided deafness.

**Conclusion:**

We show step by step how to place the new active transcutaneous bone conduction implant, Cochlear™ Osia^®^ 2 System, which utilizes a piezoelectric actuator anchored to the mastoid bone through an osseointegrated screw.

## Introduction

The Cochlear™ Osia^®^ 2 System (Osia; Cochlear, Sydney, Australia) is an active transcutaneous bone-anchored hearing implant with a newly developed piezoelectric transducer that is fixed to a titanium implant (BI300). It uses digital piezoelectric stimulation to bypass non-functional areas of the natural hearing system and send sound directly to the cochlea. This implant is designed to meet the needs of patients with unilateral and bilateral conductive or mixed hearing loss and single-sided deafness [1, 2].

Early experiences with the Osia bone conduction implant (BCI) have shown favorable results in adults. In fact, it was shown that digital piezoelectric stimulation delivers improved high frequency gain for optimizing speech perception, and maintains safety while providing excellent patient satisfaction [2–4].

The aim of this video is to show step by step how to place this new active transcutaneous BCI, which utilizes a piezoelectric actuator anchored to the mastoid bone through an osseointegrated screw.

## Methods

This device uses a transcutaneous connection between an external sound processor and an osseointegrated implant (BI300) that generates vibrations using a piezoelectricity-based internal bone-conduction system.

Before the procedure, a thorough counselling was performed with the patient and a written informed consent was obtained.

The research was conducted in accordance with the ethical principles originating in the Declaration of Helsinki.

During the surgery, it has been followed step by step the manufacturer’s recommendations.

## Results

This implant improves high frequency gain for optimal speech perception, and maintains safety and engenders high patient satisfaction.

The surgical procedure is reliable, quick and safe, the average surgical time reported in early studies is about 52 min and few surgical complications have been encountered [3]. The surgical approach used to implant the Cochlear™ Osia^®^ 2 System combines the surgical procedures employed for the conventional devices Baha Attract System and cochlear implants. While a larger incision and more soft tissue dissection is required for Osia^®^ 2 System placement, basic steps of bone fenestration, widening, polishing adjacent bone, and placing the implant are all familiar to anyone having performed traditional Baha surgery.

## Discussion

Bone conduction implants (BCIs) stimulate the cochlea directly through the vibration of the temporal bone and may be used to treat patients with unilateral and bilateral conductive or mixed hearing loss and single-sided deafness. These implants can be categorized as either percutaneous, passive transcutaneous, or active transcutaneous [2].

Percutaneous devices provide good audiometric outcomes but carry the disadvantages of poor cosmetic results and risk of skin-related complications. In contrast, the skin overlying the transcutaneous BCIs remains intact, thus decreasing the potential incidence of infections, skin reaction, and implant extrusions [2].

In October 2020, the new Osia system Osia 2 comprising the OSI200 implant and the Osia 2 sound processor (SP) was introduced by Cochlear. Since October 2020, the SP was upgraded from Osia 1 to Osia 2 SP in Freiburg. The first implantation of the new implant OSI200 in Europe was done in April 2021 [1, 5]. The Cochlear™ Osia^®^ 2 System is a new active transcutaneous BCI. The device OSI200 utilizes a piezoelectric actuator anchored to the mastoid bone through an osseointegrated screw (BI300 implant) [2].

The piezoelectric property of the actuator allows it to generate mechanical forces such as bending or vibration in response to an electrical charge. The implanted component is activated through an external SP that is attached magnetically [2].

### Surgical Procedure

The surgery is performed under general anesthesia and follows the manufacturer’s recommendations [1–3].

**Fig. 1:**
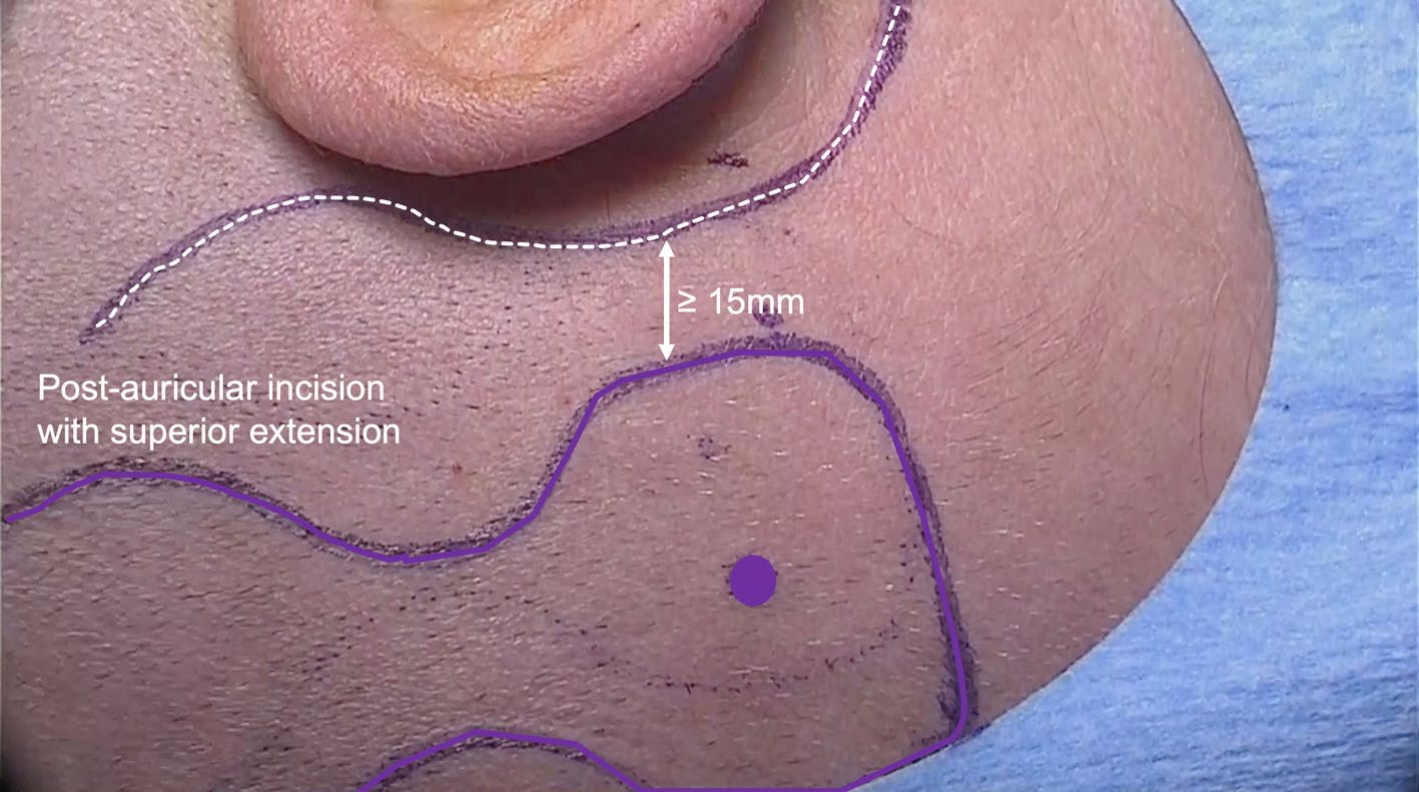
Marking the planned incision and the site for the OSI200 and the BI300 Implant. A distance of at least 15 mm between the incision and the edge of the implant can avoid skin tension and reduce the risk for post-surgical complications

**Fig. 2:**
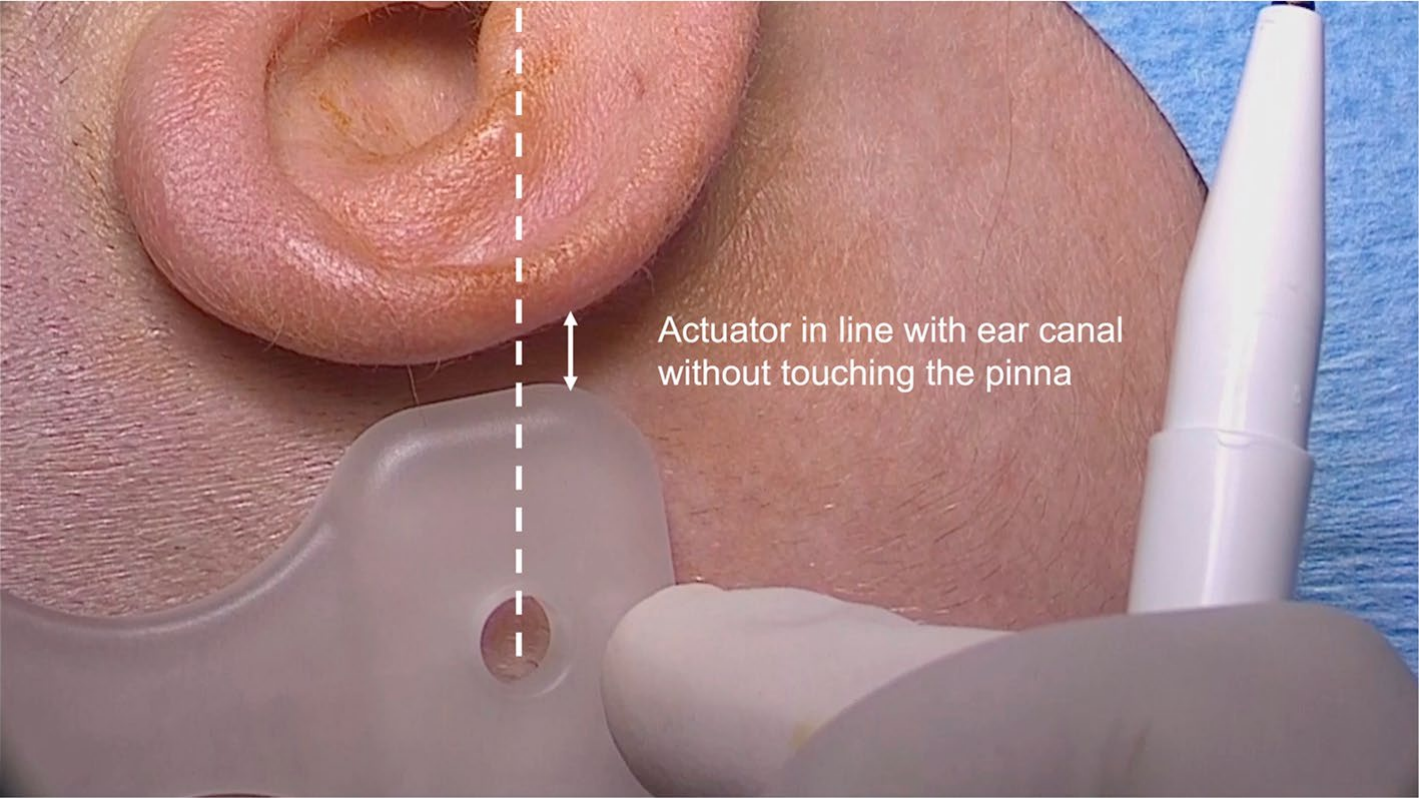
OSI200 Implant position is most optimal with the actuator in a horizontal line with the ear canal without touching the pinna

The first step is to mark the planned incision and position of the OSI200 and of the BI300 on the skin (Fig. 1). The Implant template is used to mark the shape of the OSI200 and the BI300 position. The OSI200 implant position is most optimal with the actuator in a horizontal line with the ear canal without touching the pinna (Fig. 2).

A distance of at least 15 mm between the incision and the edge of the implant can avoid skin tension and reduce the risk for post-surgical complications.

**Fig. 3:**
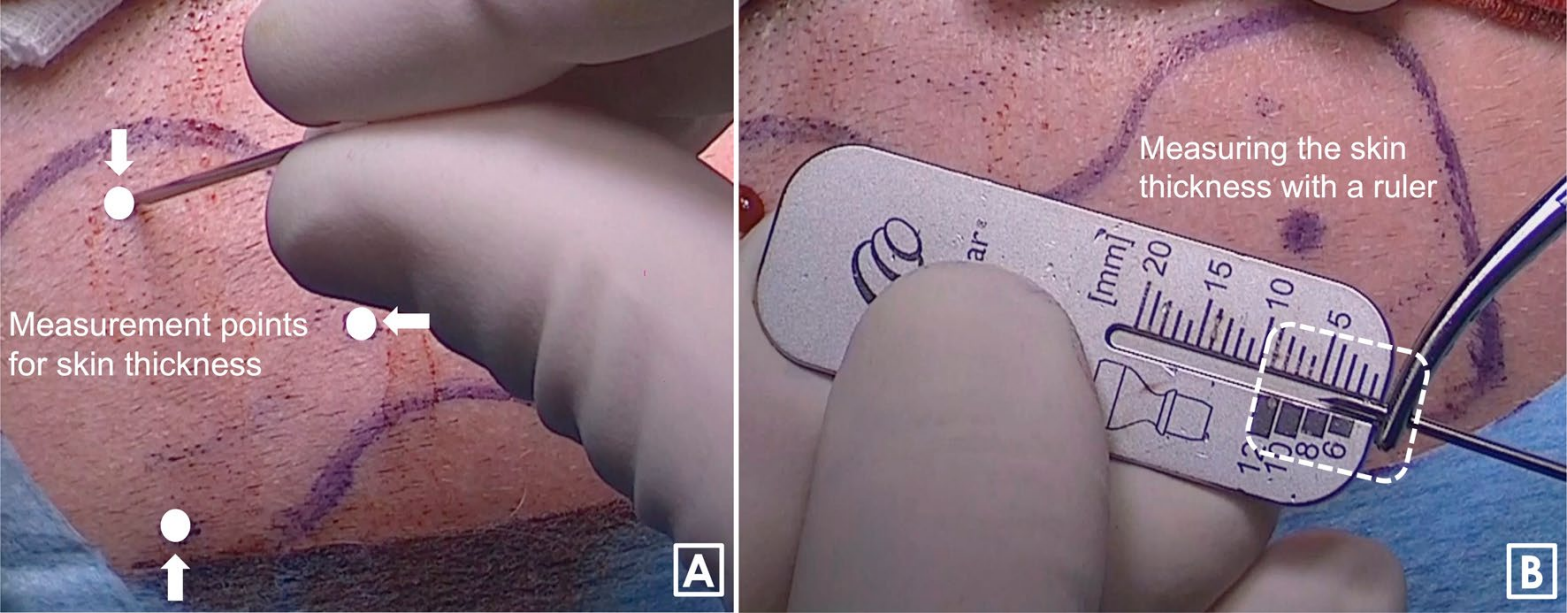
Measurement points for skin thickness (**A**). The transmitting range of the OSI200 is from 1 to 10 mm. However, the maximum skin flap thickness over the coil area is 9 mm for good magnet retention (**B**)

Then, the thickness of the soft tissue must be measured using a hypodermic needle, a clamp and a ruler. Measurement points are distributed over the coil area (Fig. 3A). The transmitting range of the OSI200 is from 1 to 10 mm. However, the maximum skin flap thickness over the coil area is 9 mm for good magnet retention (Fig. 3B).

There are three possible incision option (post-auricular incision with superior or inferior extension and posterior C-shape incision), the most commonly used incision in early studies is the post-auricular with superior extension [3].

The incision is performed and the pocket for the coil is created using blunt dissection.

A sterile OSI200 template is used to check the size of the coil pocket and the right position.

**Fig. 4:**
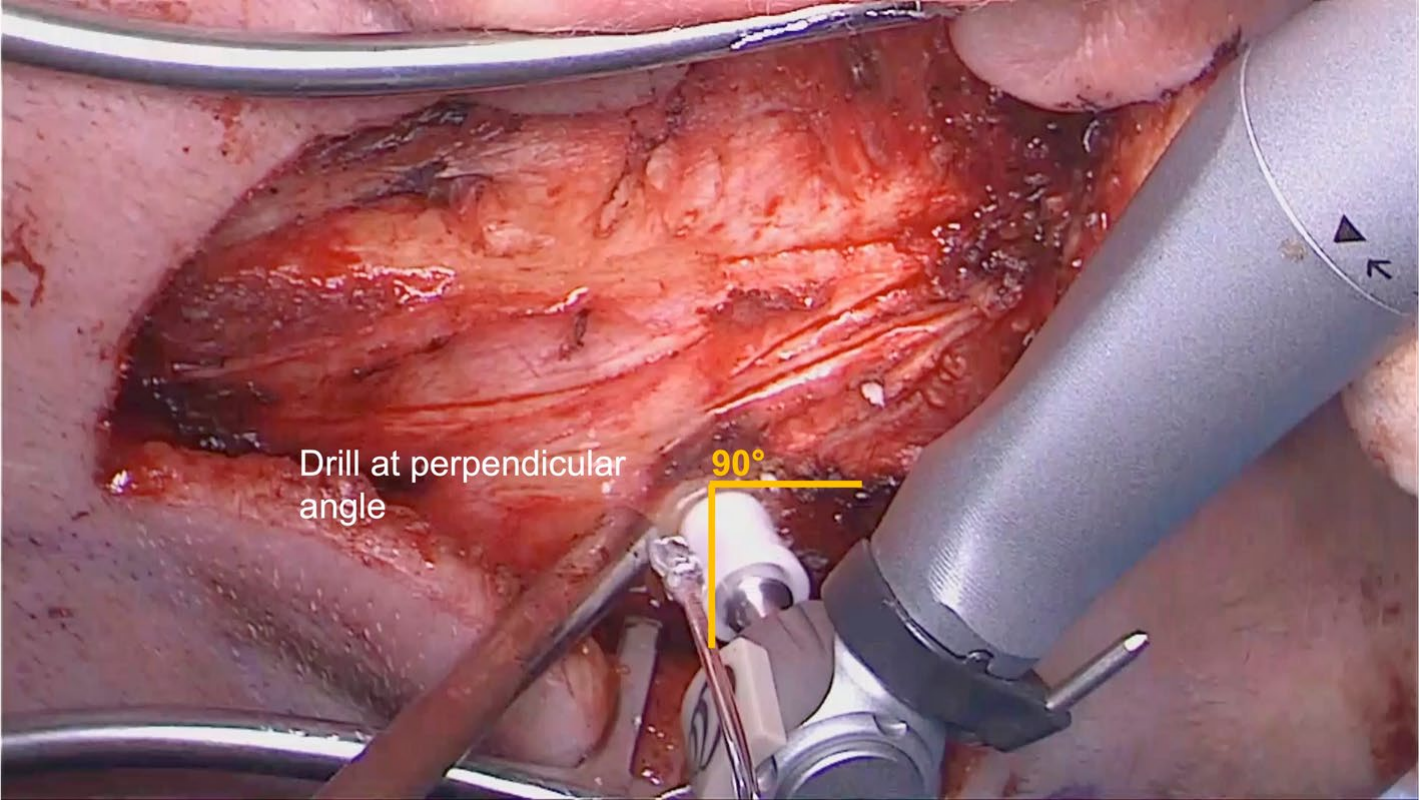
It is necessary to drill at an angle perpendicular to the bone surface to minimize the need for bone polishing later in the procedure

The periosteum is cleared away around the BI300 location, using a small cruciate incision. It is then necessary to start drilling with the conical guide drill with the 3 mm spacer at 2000 rpm. One must be sure to drill at an angle perpendicular to the surface of the bone to minimize the need to polish the bone during the surgery (Fig. 4). Intraoperatively, the thickness of the bone determined if a 3 or a 4 mm BI300 implant would be used. If there is adequate bone thickness, the white spacer on the guide drill is removed and the bone is drilled to a depth of 4 mm. The site is then widened with the widening drill and the correspondent 3 or 4 mm BI300 inserted.

The BI300 implant is picked up using the implant inserter. With the drill indicator in place, the implant is inserted at an angle perpendicular to the bone surface.

Before inserting the OSI200, the clearance indicator is used to check for interfering bone. If the bone bed indicator only touches periosteum, the periosteum must be removed. If the bone bed indicator touches bone, excess bone must be polished. It is important to provide sufficient clearance to the bone, to ensure a good connection between the actuator and the BI300.

**Fig. 5:**
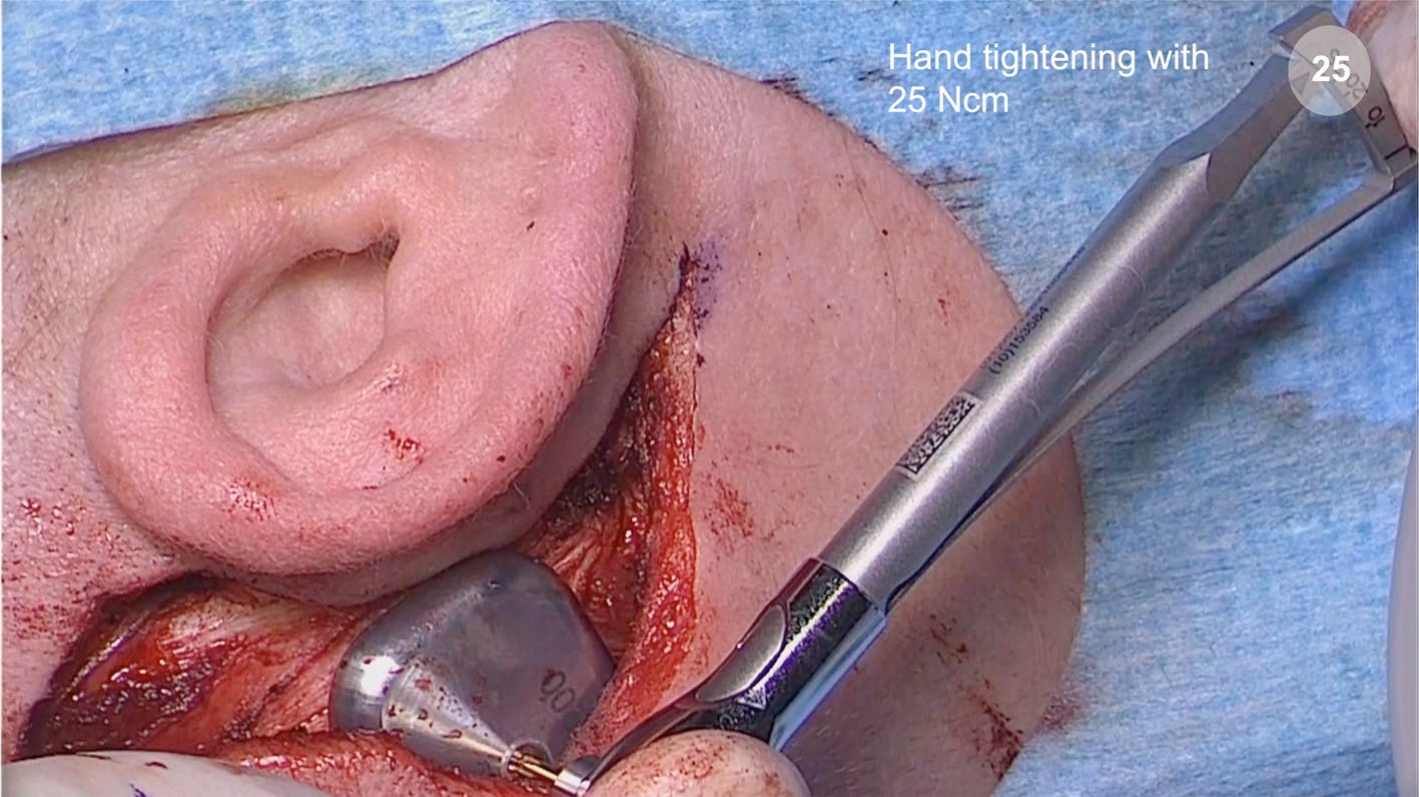
Center of the OSI200 Implant actuator is placed on top of the BI300 Implant and the fixation screw is gently hand-tightened with the screwdriver to 25 Ncm

Then, the center of the OSI200 implant actuator is placed on top of the BI300 implant and the fixation screw is gently hand-tightened with the screwdriver to 25 Ncm (Fig. 5). Following implantation, the skin flap is closed over the implant using multi-layer sutures.

In conclusion, the Cochlear™ Osia^®^ 2 System is a new active transcutaneous BCI, which represents an important advance in hearing implant technology. Being able to hear better in daily listening conditions is the ultimate goal of any hearing device, and in line with this goal, this innovative system has the capacity to allow better hearing from the day the sound processor is activated and keep patients hearing even as their needs change over time. Early experiences have shown favorable results in adults. In fact, it is safe and effective, improving speech-recognition in quiet and in noise, at low and high frequencies, thus engendering high patient hearing satisfaction [3, 4]. The surgical procedure is quick and safe: according to initial studies, the average operation time is about 52 min and few complications have been reported [3, 4]. While a larger incision and more soft tissue dissection is required for this new active transcutaneous BCI, main steps are all familiar to anyone having performed traditional Baha surgery.

It is important to follow the manufacturer’s instructions step by step to maximize the auditory results of the implant while ensuring patient comfort with a good aesthetic outcome.

## Summary

1. Bone conduction implants (BCIs) stimulate the cochlea directly through the vibration of the temporal bone.
2. They may be used to treat patients with unilateral and bilateral conductive or mixed hearing loss and single-sided deafness.
3. Unlike percutaneous BCIs, the skin overlying the transcutaneous BCIs remains intact, thus decreasing the potential incidence of infections, skin reaction, and implant extrusions.
4. The Cochlear™ Osia^®^ 2 System is a new active transcutaneous BCI.
5. The device OSI200 utilizes a piezoelectric actuator anchored to the mastoid bone through an osseointegrated screw (BI300 implant).
6. Early experiences have shown favorable results in adults.
7. It is safe and effective, improving speech-recognition in quiet and in noise, at low and high frequencies, thus engendering high patient hearing satisfaction.
8. The surgical procedure is quick and safe: according to initial studies, the average operation time is about 52 min and few complications have been reported.
9. While a larger incision and more soft tissue dissection is required for this new active transcutaneous BCI, main steps are all familiar to anyone having performed traditional Baha surgery.
10. It is important to follow the manufacturer's instructions step by step to maximize the auditory results of the implant, while ensuring patient comfort with a good aesthetic outcome.
